# Editorial: Generation of Neurons and Their Integration in Pre-existing Circuits in the Postnatal Brain: Signalling in Physiological and Regenerative Contexts

**DOI:** 10.3389/fcell.2020.00560

**Published:** 2020-07-21

**Authors:** Helena Mira, Ruth Diez del Corral, Aixa V. Morales

**Affiliations:** ^1^Instituto de Biomedicina de Valencia, CSIC, Valencia, Spain; ^2^Champalimaud Research, Champalimaud Centre for the Unknown, Lisbon, Portugal; ^3^Instituto Cajal, CSIC, Madrid, Spain

**Keywords:** adult neurogenesis, hippocampus, SVZ, neural circuits, mouse, zebrafish

During the development of the nervous system, the production of hundreds of subtypes of neurons and glial cells relies upon the relatively fast generation, amplification, specification, and differentiation of neural progenitors and neural stem cells (NSCs). This strategy, although restricted, is retained in specific niches in the adult nervous system throughout lifetime under physiological conditions. Furthermore, damage to the neural tissue in certain circumstances triggers neurogenesis leading to regeneration.

Many of the signaling cascades governing neurogenesis and circuit formation during embryogenesis are harnessed by the adult brain niches, although their regulation is substantially different in the postembryonic context. The progression from neural progenitors to differentiated new neurons must adapt to a new context with novel states emerging such as the ability of adult progenitors to enter quiescence. In addition, newly born neurons differentiate and establish their connections in an already functional network where the signals that were used to guide circuit formation during development are no longer present.

In this Research Topic, we include both original and review articles covering essential aspects of the generation and maintenance of the most prominent neurogenic niches both in teleost and in mammals such as the optic tectum in zebrafish and the hippocampal dentate gyrus (DG), the ventricular-subventricular zone (V-SVZ), and the hypothalamus in rodents. The most relevant cellular populations in those niches [neural stem cells (NSC) and immature neurons] are discussed, as well as their regulation by intrinsic programs (such as cell cycle components) and extrinsic signals such as extracellular matrix (ECM). Later steps, crucial for the integration of new neurons in existing circuits, have also been covered.

Morales and Mira review the developmental origin of the quiescent pool of adult radial glia-like NSCs with a specific focus on the hippocampal DG niche. They furthermore discuss recent work unveiling the molecular program that regulates the establishment and maintenance of the quiescent NSC state in the rodent brain. ECM signals and adhesion molecules also play a fundamental role in the regulation of the quiescence-proliferation balance and in the maintenance of NSC stemness. These aspects and the functional and structural relevance of “fractones,” a specific feature of the V-SVZ niche ECM, are thoroughly discussed in Morante-Redolat and Porlan.

Urbach and Witte summarize the available evidence on the role of the cell cycle machinery in the integration of extrinsic signals during adult hippocampal neurogenesis. They also revisit the impact of cell cycle length on the proliferation vs. differentiation decisions that take place along the neurogenic process and provide interesting insight into alternative mechanisms of fate regulation during the G1 phase of the cell cycle.

In the context of the newly discovered hypothalamic niche in the mouse, van Lingen et al. examine *Tph2* mutant mice to show that serotonin, a neuromodulator related to several homeostatic functions such as food consumption, is required to maintain appropriate levels of neurogenesis. Thus, they demonstrate an age-dependent decline in cell proliferation in the hypothalamus of Tph2 mutant mice, not observed in control mice.

Rodríguez-Iglesias et al. offer a very integrative view of what is known about the different stages followed by newborn granule cells during their maturation and integration into the hippocampal circuit in rodents and address the main functions of microglial cells in that context. Direct microglia-neuron interactions and the role of released soluble factors in the structural and functional maturation of newborn neurons are discussed.

The elucidation of the general mechanisms of circuit assembly following neurogenesis, benefits from the study of neurogenesis in non-mammalian species, which offer the advantages of tracking the process *in vivo*. Boulanger-Weill and Sumbre review recent findings showing that newborn neurons in the optic tectum of the zebrafish larva require sensory inputs for their integration into local networks and survival, reinforcing the idea that both activity-dependent and hard-wired mechanisms are involved in proper circuit integration.

Understanding the normal physiology of neurogenesis and circuit formation, has important implications in the design of therapeutic strategies aiming to promote neurogenesis and the proper wiring of new neurons in pathological situations associated to neuronal loss, including degenerative diseases and nervous system injuries.

Temporally and spatially dysregulated neuronal activity in the mouse hippocampus upon seizure induction can alter the levels of neurogenesis and induce the conversion of NSCs into reactive-like astroglial cells. However, the methods of induction of seizures that rely on intrahippocampal injections of kainic acid could be directly affecting NSCs. By using kainic acid injection in the amygdala and analyzing the effects in the hippocampus Muro-García et al. show how the reduction of neurogenesis and the reactivity of NSCs upon seizure can occur by a mechanism that depends on the surrounding neuronal circuit activity.

The possibility to manipulate specific signaling cascades to potentiate endogenous neurogenesis in cases of neuronal damage is one of the promising approaches. Geribaldi-Doldán et al. review the evidence for a role of the Protein Kinase C (PKC) pathway in regulating neurogenesis and the identification of compounds that act on specific PKC isoforms that may serve to develop new drugs acting as pro-neurogenic without affecting proliferation of other tissues.

Another pathway that enhances proliferation and neurogenesis in some contexts, including the zebrafish brain and human astrocyte cultures, is the interleukin-4/STAT6 signaling. Mashkaryan et al. explore the possibility that this role can be extended to the dentate gyrus in an Alzheimer's disease mouse model and find that, actually, this endogenous *in vivo* context behaves as a non-permissive environment with a negative effect of IL4 signaling on astroglial survival and neurogenic properties, highlighting the need to consider each context in detail.

Context seems also important in the control of neurite growth during regeneration of functional connections. By following the growth of neurites in retina bipolar cells during regeneration after injury in the adult zebrafish, McGinn et al. identify differences with the process taking place during embryonic development but also leave open the possibility that it may be sufficiently robust to restore visual function.

Finally, environmental enrichment including the combination of increased physical activity, constant cognitive stimulation, and higher social interaction, stands out as a potent regulator of adult neurogenesis in the hippocampus. Moreno-Jiménez et al. identify a major contribution of social stimuli to the promotion of neurogenesis, characterized by an increased number of newborn neurons and higher morphological maturation of their dendritic arbors.

Overall, this special issue provides an updated picture of the field and highlights the importance of the interplay between intrinsic programs and niche signals. Together, they underlie the relative plasticity of neurogenesis and may constitute key targets for regeneration.

## Author Contributions

RD and AM were the initial Guest editors of this Research Topic, inviting co-editor HM working with them to define the subjects to be treated. The three of them identified and invited leaders in specific research fields to contribute their work to the Research Topic. They acted as handling editors of manuscripts in the topic (except for Morales and Mira). They wrote the Editorial in coordination.

## Conflict of Interest

The authors declare that the research was conducted in the absence of any commercial or financial relationships that could be construed as a potential conflict of interest.

